# Nutritional priorities to support GLP‐1 therapy for obesity: A joint Advisory from the American College of Lifestyle Medicine, the American Society for Nutrition, the Obesity Medicine Association, and The Obesity Society

**DOI:** 10.1002/oby.24336

**Published:** 2025-05-30

**Authors:** Dariush Mozaffarian, Monica Agarwal, Monica Aggarwal, Lydia Alexander, Caroline M. Apovian, Shagun Bindlish, Jonathan Bonnet, W. Scott Butsch, Sandra Christensen, Eugenia Gianos, Mahima Gulati, Alka Gupta, Debbie Horn, Ryan M. Kane, Jasdeep Saluja, Deepa Sannidhi, Fatima Cody Stanford, Emily A. Callahan

**Affiliations:** ^1^ Food is Medicine Institute, Friedman School of Nutrition Science and Policy Tufts University Boston Massachusetts USA; ^2^ Division of Endocrinology, Diabetes and Metabolism, Department of Medicine University of Alabama at Birmingham Birmingham Alabama USA; ^3^ Division of Cardiology University of Florida Gainesville Florida USA; ^4^ Enara Health San Mateo California USA; ^5^ Center for Weight Management and Wellness, Division of Endocrinology, Diabetes and Hypertension, Department of Medicine Brigham and Women's Hospital, Harvard Medical School Boston Massachusetts USA; ^6^ Department of Medicine Touro University and One Medical Dublin California USA; ^7^ Division of Primary Care and Population Health Stanford University School of Medicine Palo Alto California USA; ^8^ Department of Surgery Bariatric and Metabolic Institute, Cleveland Clinic Lerner College of Medicine, Cleveland Clinic Cleveland Ohio USA; ^9^ Integrative Medical Weight Management Seattle Washington USA; ^10^ Northwell Cardiovascular Institute Lenox Hill Hospital New Hyde Park New York USA; ^11^ Division of Endocrinology, Diabetes, and Metabolism, Department of Medicine University of Connecticut Health Farmington Connecticut USA; ^12^ Division of General Internal Medicine Weill Cornell Medicine New York New York USA; ^13^ Division of General Internal Medicine George Washington University Washington DC USA; ^14^ Center for Obesity Medicine and Metabolic Performance University of Texas at Austin Austin Texas USA; ^15^ Division of General Internal Medicine, Department of Medicine Duke University Durham North Carolina USA; ^16^ Clinical and Translational Science Institute Duke University Durham North Carolina USA; ^17^ Aroga Lifestyle Medicine Victoria British Columbia Canada; ^18^ Department of Family Medicine University of California San Diego San Diego California USA; ^19^ Department of Medicine‐Division of Endocrinology‐Neuroendocrine Massachusetts General Hospital, MGH Weight Center Boston Massachusetts USA; ^20^ Department of Pediatrics‐Division of Endocrinology Nutrition Obesity Research Center at Harvard (NORCH) Boston Massachusetts USA

## Abstract

**Background:**

Glucagon‐like peptide 1 receptor agonists and combination medications (hereafter collectively referred to as GLP‐1s) are shifting the treatment landscape for obesity. However, real‐world challenges and limited clinician and public knowledge on nutritional and lifestyle interventions can limit GLP‐1 efficacy, equitable results, and cost‐effectiveness.

**Objectives:**

We aimed to identify pragmatic priorities for nutrition and other lifestyle interventions relevant to GLP‐1 treatment of obesity for the practicing clinician.

**Methods:**

An expert group comprising multiple clinical and research disciplines appraised the scientific literature, informed by expert knowledge and clinical experience, to identify and summarize relevant topics, priorities, and emerging directions.

**Results:**

GLP‐1s reduce body weight by 5% to 18% in trials, with modestly lower effects in real‐world analyses, and multiple demonstrated clinical benefits. Challenges include side effects, especially gastrointestinal; nutritional deficiencies due to calorie reduction; muscle and bone loss; low long‐term adherence with subsequent weight regain; and high costs with resulting low cost‐effectiveness. Numerous practice guidelines recommend multicomponent, evidence‐based nutritional and behavioral therapy for adults with obesity, but use of such therapies with GLP‐1s is not widespread. Priorities to address this include: (a) patient‐centered initiation of GLP‐1s, including goals for weight reduction and health; (b) baseline screening, including usual dietary habits, emotional triggers, disordered eating, and relevant medical conditions; (c) comprehensive exam including muscle strength, function, and body composition assessment; (d) social determinants of health screening; (e) and lifestyle assessment including aerobic activity, strength training, sleep, mental stress, substance use, and social connections. During GLP‐1 use, nutritional and medical management of gastrointestinal side effects is critical, as is navigating altered dietary preferences and intakes, preventing nutrient deficiencies, preserving muscle and bone mass through resistance training and appropriate diet, and complementary lifestyle interventions. Supportive strategies include group‐based visits, registered dietitian nutritionist counseling, telehealth and digital platforms, and Food is Medicine interventions. Drug access, food and nutrition insecurity, and nutrition and culinary knowledge influence equitable obesity management with GLP‐1s. Emerging areas for more study include dietary modulation of endogenous GLP‐1, strategies to improve compliance, nutritional priorities for weight maintenance post‐cessation, combination or staged intensive lifestyle management, and diagnostic criteria for clinical obesity.

**Conclusions:**

Evidence‐based nutritional and lifestyle strategies play a pivotal role to address key challenges around GLP‐1 treatment of obesity, making clinicians more effective in advancing their patients' health.

## INTRODUCTION

With high and rising rates of adiposity and related morbidity, mortality, and healthcare expenditures, recently approved glucagon‐like peptide 1 receptor agonists and related combination obesity medications are shifting the treatment landscape (we collectively refer to these as “GLP‐1s” given this common practical usage by clinicians, policy makers, and the public; we acknowledge the lack of any widely accepted terminology to describe this new class of obesity medications). In randomized trials, GLP‐1s produce placebo‐adjusted weight reduction of 5% to 18% among individuals with obesity or overweight and weight‐related complications. This efficacy has generated enormous attention and utilization [1]. In 2024, 6% of United States (US) adults report current GLP‐1 use, and 12% report current or past use—rising to 22% among individuals told by a clinician that they have overweight or obesity [2].

Despite the efficacy and growing utilization of these medications, real‐world challenges are increasingly evident [3]. These include gastrointestinal (GI) side effects; risk of inadequate nutrient intake from reduced food intake combined with insufficient nutritional counseling; potential loss of significant muscle mass and bone density [4]; high discontinuation rates (e.g., 50%–67% at 1 y and 85% at 2 years [5, 6, 7, 8]) that may relate to side effects, costs, variable individual efficacy, or patient preferences [5, 9]; and limited public and clinician knowledge on the importance and implementation of complementary nutritional and lifestyle changes.

All these challenges may be partially mitigated by an evidence‐based, structured lifestyle program, particularly around food, when prescribing GLP‐1s for obesity. However, practical guidance for clinicians to implement such an approach is limited. This Advisory combines expertise across clinical and research societies focused on obesity, lifestyle, and nutrition to provide such guidance. It addresses current topics of interest among patients and clinicians, summarizes uncertainties, and highlights future research directions. Although the general focus is on the US context, the recommendations have implications for use of GLP‐1s for obesity management globally.

## OVERVIEW OF EFFICACY, SIDE EFFECTS, AND KEY CHALLENGES

GLP‐1 receptor agonists such as semaglutide and liraglutide, as well as combination agents like tirzepatide (which adds glucose‐dependent insulinotropic polypeptide receptor agonism)—all hereafter referred to as GLP‐1s for brevity—are effective new agents for obesity treatment which demonstrate weight reduction, weight maintenance, and reduced morbidity and mortality. These medications are approved by the Food and Drug Administration (FDA) for the treatment of obesity or overweight with weight‐related comorbidities. Semaglutide and liraglutide are indicated for adults or youth aged ≥12 years and tirzepatide for adults aged ≥18 years [10, 11, 12]. GLP‐1s for obesity have additional FDA‐approved indications for cardiovascular disease risk reduction (semaglutide) and obstructive sleep apnea (tirzepatide). GLP‐1s are separately approved for type 2 diabetes and chronic kidney disease, which are not the focus of this Advisory.

### Efficacy

In the original phase 3 randomized trials for obesity, average weight reduction compared to placebo has ranged from 5.3% to 17.8% after 56 to 72 weeks (Table 1), with improvements in several obesity‐related risks and complications [13, 14, 15]. In real‐world practice, the efficacy for weight reduction is often lower, for example, about 8% for individuals with diabetes and 11% for individuals without diabetes at 60 weeks. with semaglutide 2.4 mg/day [4]. Generally, weight reduction is more rapid during the first 6 months and slows thereafter, with relative plateauing at 18 months [13, 14, 15]. When GLP‐1 use is continued, weight reduction is sustained for at least 4 years in controlled and observational studies [16, 17].

**TABLE 1:** Efficacy and outcomes at 52 weeks of GLP‐1 therapy^a^ in the landmark industry‐sponsored randomized controlled trials.

Medication	Mean intervention weight reduction	Mean placebo weight reduction	Mean placebo‐adjusted GLP‐1 effect	Metabolic risk and health outcomes improved	Key exclusion criteria
Liraglutide 3.0 mg/week^b^	7.9%	2.6%	5.3%	Glycemic control (glycated hemoglobin, fasting glucose, fasting insulin), systolic and diastolic blood pressure, cholesterol (total, LDL, HDL, VLDL, non‐HDL), triglycerides, free fatty acids, health‐related quality of life	Type 1 or 2 diabetes Use of medications that cause clinically significant weight gain or loss Previous bariatric surgery Personal history of pancreatitis; major depressive or other severe psychiatric disorders Personal or family history of multiple endocrine neoplasia type 2 or familial medullary thyroid carcinoma
Semaglutide 2.4 mg/week^c^	14.9%	2.4%	12.4%	Waist circumference, systolic blood pressure, physical functioning scores	History of type 1 or type 2 diabetes mellitus Glycated hemoglobin ≥6.5% Personal history of chronic pancreatitis, acute pancreatitis within 180 days before enrollment Previous surgical treatment for obesity Treatment with a medication that promotes weight loss within 90 days before enrollment
Tirzepatide 5 mg/week^d^	15.0%	3.1%	11.9%	Waist circumference, systolic and diastolic blood pressure, physical functioning scores, triglycerides, cholesterol (total, LDL, VLDL, HDL, non‐HDL), free fatty acids, fasting insulin	History of type 1 or type 2 diabetes mellitus Change in body weight >5 kg within 90 days before screening Previous or planned surgical treatment for obesity Treatment with a medication that promotes weight loss within 90 days before screening
Tirzepatide 10 mg/week^d^	19.5%	3.1%	16.4%	As above	As above
Tirzepatide 15 mg/week^d^	20.9%	3.1%	17.8%	As above	As above

^a^ Includes tirzepatide, which combines GLP‐1 agonism with glucose‐dependent insulinotropic polypeptide receptor agonism.

^b^ Data from reference [13], primary outcomes at 56 weeks.

^c^ Data from reference [14], primary outcomes at 68 weeks.

^d^ Data from reference [15], primary outcomes at 72 weeks.

When GLP‐1s are discontinued, weight regain is common—with up to two‐thirds of the lost weight regained within 1 years [18, 19, 20]. Notably, this has been observed even with accompanying use of conventional nutritional counseling and/or behavioral therapy [18, 19]. The potential for more robust, structured nutrition and lifestyle therapy to mitigate weight gain after GLP‐1 cessation has not been studied in controlled trials.

Although these findings describe the average response, individual responses can vary widely, highlighting the complexity of obesity as a disease. Some people experience minimal weight reduction with GLP‐1s, whereas others have robust weight reductions of ≥30%. In multivariate analyses, factors predicting larger responses with tirzepatide include female sex (2.4 higher odds of achieving a 20% weight reduction), lower baseline hemoglobin A1c (1.62 higher odds), no diagnosed hypertension (1.35 higher odds), and lower ALT (1.17 higher odds) [21]; and in univariate (crude) analyses with semaglutide, female sex (48% greater weight loss in kilograms), younger age (24% greater for age <55 vs. ≥75 years), and higher baseline BMI (23% greater for ≥40 vs. <30 kg/m^2^) [16].

Demonstrated clinical benefits of GLP‐1s include improved cardiometabolic risks, fewer major adverse cardiovascular events [22], decreased mortality in heart failure [23, 24, 25, 26, 27], and improvements in obstructive sleep apnea [21, 28], prediabetes [29, 30], chronic kidney disease [31], knee osteoarthritis [32], substance use disorders [33], and metabolic‐associated steatotic liver disease [34]. Trials have been conducted for other outcomes, such as breast cancer and neurodegenerative disorders [35, 36, 37]. Although many of these benefits are weight‐dependent, others appear at least partly weight‐independent. For example, hemoglobin A1c reduction can occur without weight change, and reduced risk of cardiovascular events appears to emerge before substantial weight reduction [38, 39].

### Side effects

Side effects are relatively common but usually not severe. These are more likely to occur within the first weeks of initiation of therapy and with dose escalation. Side effects tend to decrease in frequency and severity with continuation of a stable dose [40]. GI side effects are most frequent and include nausea (25%–44%), diarrhea (19%–30%), vomiting (8%–24%), constipation (17%–24%), and abdominal pain (9%–20%) [41, 42, 43, 44, 45, 46]. Although certain side effects have been reported more commonly with semaglutide than with tirzepatide, trial data suggest that such differences may be a reflection of variation in background (i.e., placebo group) rates in the enrolled trial populations, with the proportional increase in many side effects when compared to placebo being similar for the 2 agents (Table 2). Emerging therapies, such as dual and triple receptor agonists targeting GLP‐1, glucose‐dependent insulinotropic polypeptide (GIP), and glucagon pathways, aim to improve efficacy while reducing GI side effects [44]. Real‐world experience has largely mirrored these trial data, with GI issues (nausea, diarrhea, vomiting, constipation) being the most frequently reported side effects [47, 48].

**TABLE 2:** Common side effects reported in semaglutide and tirzepatide trials for obesity^a^.

Side effect	Semaglutide 2.4 mg group (%)	Placebo group (%)	Tirzepatide 15 mg group (%)	Placebo group (%)
Nausea	44	16	28	8
Diarrhea	30	16	23	8
Vomiting	24	6	13	2
Constipation	24	11	11	5
Abdominal pain	20	10	10	5
Headache	14	10	‐	‐
Fatigue	11	5	7	3
Dyspepsia	9	3	10	4
Dizziness	8	4	4	2
Abdominal distension	7	5	4	2
Eructation	7	<1	5	1
Hypoglycemia^b^	6	2	‐	‐
Flatulence	6	4	4	2
Gastroenteritis	6	4	‐	‐
Gastroesophageal reflux	5	3	5	2
Gastritis	4	1	‐	‐
Hair loss	3	1	5	1

^a^ Data from references [42] and [43], based on follow‐up periods of up to 68 weeks (semaglutide) or 72 weeks (tirzepatide).

^b^ Among individuals with type 2 diabetes.

In the trials, GI symptoms rarely led to discontinuation, with <10% of participants stopping therapy due to GI issues [49]. Fewer data are available on the impact of GI side effects on adherence in clinical practice. Combining GLP‐1s with metformin does not appear to worsen GI side effects, despite metformin's association with similar symptoms [50].

Underlying causes of these GI symptoms appear varied. GLP‐1s delay gastric emptying, leading to bloating, fullness, and nausea [43]. GLP‐1s activate several brain regions responsible for weight regulation, appetite, and nausea [51]. Occasionally, GLP‐1s affect intestinal motility or secretions, contributing to diarrhea [46]. Higher doses are more likely to provoke these adverse symptoms, indicating a dose‐dependent relationship [40].

Less common side effects included dyspepsia, fatigue, headache, eructation (belching), hair loss, gastroesophageal reflux, dizziness, and gastritis (Table 2). Hypoglycemia can occur in individuals with type 2 diabetes, especially when they are consuming insulin or insulin secretagogues such as sulfonylureas [12, 41]. Rare side effects include gallbladder disease, pancreatitis, acute kidney injury (typically related to hypovolemia), hypersensitivity reactions, and gastroparesis [12, 41]. Ophthalmic complications have been rarely reported, which could relate to direct toxicity or rapid GLP‐1‐correction of hyperglycemia [52]. Rare cases of suicidality have been reported, although preliminary evaluation using the FDA Adverse Reporting System, post hoc analysis of the STEP clinical trials, and 1 large cohort study have not confirmed any definitive link; the FDA and European Agencies are monitoring potential risk [53, 54, 55].

### Nutritional deficiencies

Individuals using GLP‐1s to treat obesity experience significant reductions in appetite and energy intake, with observed caloric reductions of 16%–39% [56]. This large, rapid reduction can lead to insufficient intakes of essential vitamins and minerals, especially at energy intakes <1200 kcal/d for females and < 1800 kcal/d for males [57]. Some examples of nutrients of concern include iron, calcium, magnesium, zinc, and vitamins A, D, E, K, B1, B12, and C [58]. Signs of frank nutrient deficiency include fatigue beyond expected levels, excessive hair loss, skin flakiness or itching, muscle weakness, poor wound healing, and unusual bruising [59]. GI side effects may further compromise nutrient absorption and exacerbate preceding or new risk of nutrient insufficiency.

Individuals with obesity are also more likely to have suboptimal dietary patterns at baseline that predispose them to nutrient deficiencies prior to starting therapy, for example, due to high ultraprocessed food consumption or highly restrictive diets [60]. Obesity itself can also increase risk of nutrient deficiencies at baseline due to alterations in nutrient absorption, distribution, metabolism, or excretion [61]. All these issues highlight the importance of proactively managing dietary composition and quality to maximize nutrient intake within a lower calorie intake [58].

### Muscle and bone loss

Rapid weight reduction from (but not limited to) GLP‐1 use frequently leads to loss of both fat and muscle mass [62, 63]. In the STEP 1 trial, of the average 13.6‐kg–weight reduction, 8.3 kg (62%) was fat mass and 5.3 kg (38%) was lean body mass (including muscle and other nonfat tissues) [14]. Because muscle mass is about half of lean body mass, this corresponds to ~20% of total weight reduction being muscle loss. In the SURMOUNT 1 trial (pooling doses), total lean mass was reduced by 8.5 absolute percentage points [15]. Modeling data suggest that loss of muscle mass varies by sex, representing 10%–15% of total weight reduction in females and 20%–25% of total weight reduction in males, in the absence of structured strength training [64].

These reductions in fat mass, lean body mass, and muscle mass correlate with the degree of body weight reduction and are similar to those documented with other obesity therapies that achieve large weight reductions, such as bariatric surgery and very low‐calorie restricted diets [65]. However, lean mass reduction is also affected by the degree of calorie restriction, overall rapidity of weight reduction, and presence or absence of strength training exercises [66]. Low protein consumption due to reduced appetite may also contribute to muscle loss and increased risk for sarcopenia, particularly among those with older age, perimenopausal or menopausal status, lower testosterone, sedentary behavior, or lack of resistance/strength training [67, 68, 69, 70].

Rapid weight reduction with GLP‐1s or other therapies can also affect bone density. Weight reduction that is substantial (≥14%) and rapid (over 3–4 months) is associated with significant bone loss [71], whereas more moderate and slower weight reduction may better preserve bone mass [72]. Bone loss is influenced by initial body weight, age, sex, physical activity, extent of energy restriction and protein intake, and rate of weight reduction, with older individuals and females experiencing greater bone loss [71]. In the absence of structured nutrition and exercise efforts, loss of muscle and bone may be exacerbated by intermittent use of GLP‐1s and weight regain or “weight cycling,” increasing risk of sarcopenic obesity.

### Adherence and costs

In manufacturer‐sponsored trials of GLP‐1s for obesity, reported adherence (sustained use) has ranged from 83% to 88% at 66–68 weeks [15, 73]. Adherence is much lower in practice: about 33%–50% at 1 years and 15% at 2 years [5, 6, 7, 8]. Discontinuation is associated with older age (≥65 years), poor weight response, and moderate or severe GI side effects [74]. The relative influences of other factors on discontinuation are unclear, including changes in insurance coverage, high out‐of‐pocket costs, medication shortages [75], or “false cessation” due to switching to compounded (pharmacy prepared) GLP‐1s. Low adherence may also relate to lower public and clinician awareness of the need for long‐term use after a weight goal, health goal, or plateau is reached.

The current US list price for GLP‐1s for obesity ranges from ~$12,000 to $16,000 per year [2]. Full costs may be incurred by those who self‐pay, due to either off‐label use or no payer coverage. With manufacturer coupons and discounts, costs can be lowered to ~$7000 to $8000 per year [76, 77, 78]. Coverage and costs for Medicaid programs vary by state, as each state determines coverage decisions and negotiates prices with the drug manufacturers. Some states have dropped coverage for GLP‐1s due to high costs and unsuccessful pricing negotiations [79]. Medicare does not currently cover GLP‐1s for obesity, but recently announced that they will be among the drug classes which the federal government will aim to negotiate in 2025; average price reductions in prior negotiations for other drug classes have ranged from 38% to 79% from the original list price [80]. Coverage by private insurers is highly variable, with some providing coverage, others providing coverage but with clinical restrictions or lifetime caps, and others not providing coverage. Local and regional compounding pharmacies also directly manufacture GLP‐1s, with gray literature prices from ~$1800 to $3000 per year [81]. However, this is not regulated by the FDA for safety or efficacy; and recent FDA guidance has aimed to eliminate this production.

Several studies have estimated the cost‐effectiveness of GLP‐1s for obesity from a healthcare perspective, considering costs for screening and treatment against savings from improved weight and health outcomes and corresponding long‐term reductions in healthcare utilization, including downstream accumulated health benefits. All have found that GLP‐1 treatment costs exceed healthcare savings. In one analysis, individuals with obesity treated with GLP‐1s incurred significantly higher annual healthcare costs than individuals with obesity without GLP‐1 use (~$7000 higher in the first year, and $4200 higher in the second year) [6, 82]. Considering cost‐effectiveness, that is, health gained per dollar spent, most studies find that GLP‐1s, even at currently discounted prices, do not meet accepted thresholds for cost‐effective therapy (e.g., <$150,000 per quality‐adjusted life year gained). In nonindustry‐sponsored analyses, net costs per quality‐adjusted life year have ranged from $237,000 to $483,000 [83], with low cost‐effectiveness related to plateauing of weight benefits but continued high costs of treatment, as well as weight regain following any cessation of use. These high costs, lower adherence in practice, and frequent weight regain after discontinuation, each highlight the importance of complementary nutritional and lifestyle counseling to help maximize overall efficacy and cost‐effectiveness [84].

## GUIDELINES AND PRACTICE FOR NUTRITION AND LIFESTYLE COUNSELING FOR OBESITY

The high and rising prevalence of obesity globally is often attributed to poor dietary patterns and insufficient physical activity, which are often related to behaviors learned early in life and developed over time as well as structural barriers to good lifestyle [85]. Serial studies from the US and Europe suggest that energy expenditure from physical activity increased between 1981 and 2017, during the onset of the obesity epidemic, while basal metabolic expenditure declined, implicating other factors such as dietary composition that impact metabolic rate [86]. Beyond obvious effects on energy balance, dietary quality can also influence obesity risk through changes in metabolic adaptation due to a high refined carbohydrate diet, in resting energy expenditure (such as through brown or beige adipose tissue thermogenesis), in microbiome calorie utilization (with corresponding greater or lesser utilization by the host tissues), and in epigenetic or trans‐generational risk of obesity [87, 88]. Thus, dietary composition, beyond calories alone, is relevant to obesity risk.

Although individual risk can be modified by genetic influences, population risk and trends in obesity over time are predominantly driven by lifestyle trends related to cultural, community, and environmental factors. Obesity can also be exacerbated by iatrogenic causes, resulting from poor diet quality or low physical activity due to medical conditions or obesogenic medications [89, 90]. Although all causes and contributors of obesity are not within an individual's control, structured lifestyle modification programs can be effective and feasible to help achieve a 5%–10% weight reduction and maintain a healthy body weight for many people [91, 92, 93].

### Current guidelines

The United States Preventive Services Task Force (USPSTF) has published several recommendations for multicomponent lifestyle and behavioral therapy for adults with obesity, cardiovascular disease risk factors, and prediabetes, and clinical societies have identified several evidence‐based recommendations for lifestyle modification for obesity (Table 3) [94, 95, 96, 97, 98, 99, 100]. According to the USPSTF, the evidence supports referring all adults with obesity to intensive, multicomponent behavioral interventions for both weight reduction and weight maintenance. Intervention components should include nutrition, physical activity, self‐monitoring, identifying barriers, problem solving, peer support, and relapse prevention—each further discussed in this Advisory.

**TABLE 3:** Key guidelines for lifestyle modification therapies for individuals with obesity^a^.

Organization	Recommendations
American Heart Association/American College of Cardiology/The Obesity Society (2013^b^)	Counsel overweight and obese adults with CVD risk factors (hypertension, hyperlipidemia, hyperglycemia) that lifestyle changes that produce even modest, sustained weight reduction of 3%–5% produce clinically meaningful health benefits, and greater weight reduction produces greater benefits (Grade I‐A) Prescribe a diet to achieve reduced calorie intake for weight reduction (Grade I‐A) Advise/prescribe participation in a comprehensive lifestyle intervention for 6 or more months (including at least 14 sessions over 6 months) (Grade I‐A).
United States Preventive Services Task Force (2018^c^)	Offer or refer adults with obesity to intensive, multicomponent behavioral interventions. This includes weight reduction and weight reduction maintenance interventions with components that focus on nutrition, physical activity, self‐monitoring, identifying barriers, problem solving, peer support, and relapse prevention (B recommendation).
United States Preventive Services Task Force (2020^d^)	Offer or refer adults with CVD risk factors (hypertension, dyslipidemia, metabolic syndrome, or estimated 10‐year CVD risk >7.5%) to behavioral counseling interventions to promote a healthy diet and physical activity (B recommendation).
Canadian Medical Association (2020^e^)	Adults living with obesity should receive individualized care plans that address their root causes of obesity and that provide support for behavioral change (e.g., nutrition, physical activity). Adults living with obesity should receive individualized medical nutrition therapy provided by a registered dietitian (when available) to improve weight outcomes (body weight, BMI), waist circumference, glycemic control, established lipid, and blood pressure targets. (Level 1a, Grade A) Adults living with obesity can consider any of multiple medical nutrition therapies to improve health‐related outcomes, choosing the dietary patterns and food‐based approaches that support their best long‐term adherence.
United States Preventive Services Task Force (2021^f^)	Screen adults aged 35 to 70 years who have overweight or obesity for prediabetes and diabetes; and offer or refer patients with prediabetes to effective preventive interventions, such as lifestyle interventions that focus on diet, physical activity, or both (e.g., the Diabetes Prevention Program) (B recommendation^†^).
European Association for the Study of Obesity (2024^g^)	Provide behavioral modifications for all persons with obesity, including nutritional therapy, physical activity, stress reduction, and sleep improvement.

^a^ This table presents key examples, not necessarily a complete compendium, of major lifestyle recommendations from these reports.

^b^ Data from reference [94]. Grade I‐A is an indicator of the recommendation and its level of evidence, here denoting that the procedure or treatment should be performed/administered and has strong evidence that it is useful/effective.

^c^ Data from reference [95]. B recommendation is an indicator that the USPSTF recommends the service. There is high certainty that the net benefit is moderate or there is moderate certainty that the net benefit is moderate to substantial. Suggestions for practice: Offer or provide this service.

^d^ Data from reference [96]. An update for this topic was in progress during this manuscript's development [100].

^e^ Data from reference [98].

^f^ Data from reference [97].

^g^ Data from reference [99].

Although specifics of lifestyle programming for weight reduction and maintenance vary across guidelines, common foundations include a nutrient‐dense, reduced‐calorie diet; a structured program of physical activity; and behavioral strategies to support lifestyle change [94]. Various dietary patterns have been used with success, with adherence to counseling visits and the selected diet often being the important factors in determining outcomes [101]. Specific nutrient goals can vary by age, sex, and life stage (e.g., infancy, childhood, adolescence, pregnancy, lactation, older adulthood) [102] as well as comorbidities or clinical conditions.

Based on existing guidelines and evidence, all individuals who would benefit from obesity treatment, including those prescribed GLP‐1s, should be offered or referred for intensive, multicomponent behavioral interventions for both weight reduction and weight maintenance [103]. The specific parameters can be based on patient‐centered shared decision‐making, considering each person's stage and severity of disease, risk of progression, and comorbidities; and centered on the individual's values and goals, stage of change, and access to therapies.

### Current practice

Although authoritative health and medical entities recommend comprehensive lifestyle modification as part of the treatment plan for obesity, the use of such therapies before or to support GLP‐1 use is not widespread in practice. Visits with primary care physicians and nonobesity medicine specialists who care for individuals with obesity are usually short and centered on acute illness or needs, screening discussions, and medication management [104]. In addition, access is limited to lifestyle medicine approaches for obesity and its comorbidities. For example, the Diabetes Prevention Program is known to reduce the risk of progression to diabetes and is covered by major payers, but has not been meaningfully scaled due to regulatory and implementation barriers [105, 106]. In addition, although health coaching is theoretically reimbursable by some private insurers, employee wellness benefits, Medicare Advantage plans, and state Medicaid programs, the lack of approval of category I Current Procedural Terminology codes for health coaching by the American Medical Association remains a barrier to reimbursement [107, 108]. Intensive behavioral therapy can be billed only by primary care providers [109, 110]. As discussed later in this Advisory, private and public payer coverage for medical nutrition therapy (MNT) for obesity remains limited, preventing broad utilization in practice. These pressures, alongside a frequent lack of practitioner education about integrating lifestyle management in medicine, have created a dearth of implemented behavioral and lifestyle counseling, accessible and effective referral programs, and integration into existing care delivery systems.

As GLP‐1s are becoming more commonly prescribed for obesity and other health conditions by providers across multiple disciplines, there is growing concern for the continued lack of formal medical training in nutrition and obesity and the paucity of basic knowledge and competencies to provide nutrition counseling [111, 112]. For example, one study found that 90% of cardiologists receive minimal or no nutrition education during fellowship [113]—despite the critical role of diet in cardiovascular health. Academic experts, the US House of Representatives, and clinical societies have called for reform to support and facilitate more robust nutrition education and training in US undergraduate and graduate medical education [114, 115, 116, 117, 118]. In this writing group's experience, we observe that many individuals prescribed GLP‐1s have not received meaningful nutrition or other lifestyle guidance preceding, accompanying, or (if the drug is stopped) after the therapy. The absence of such behavioral counseling can impede understanding and expectations around medication use and side effects, efficient clinical follow‐up, overall efficacy, and long‐term weight maintenance.

## NUTRITIONAL PRIORITIES TO SUPPORT GLP‐1 THERAPY

**FIGURE 1:**
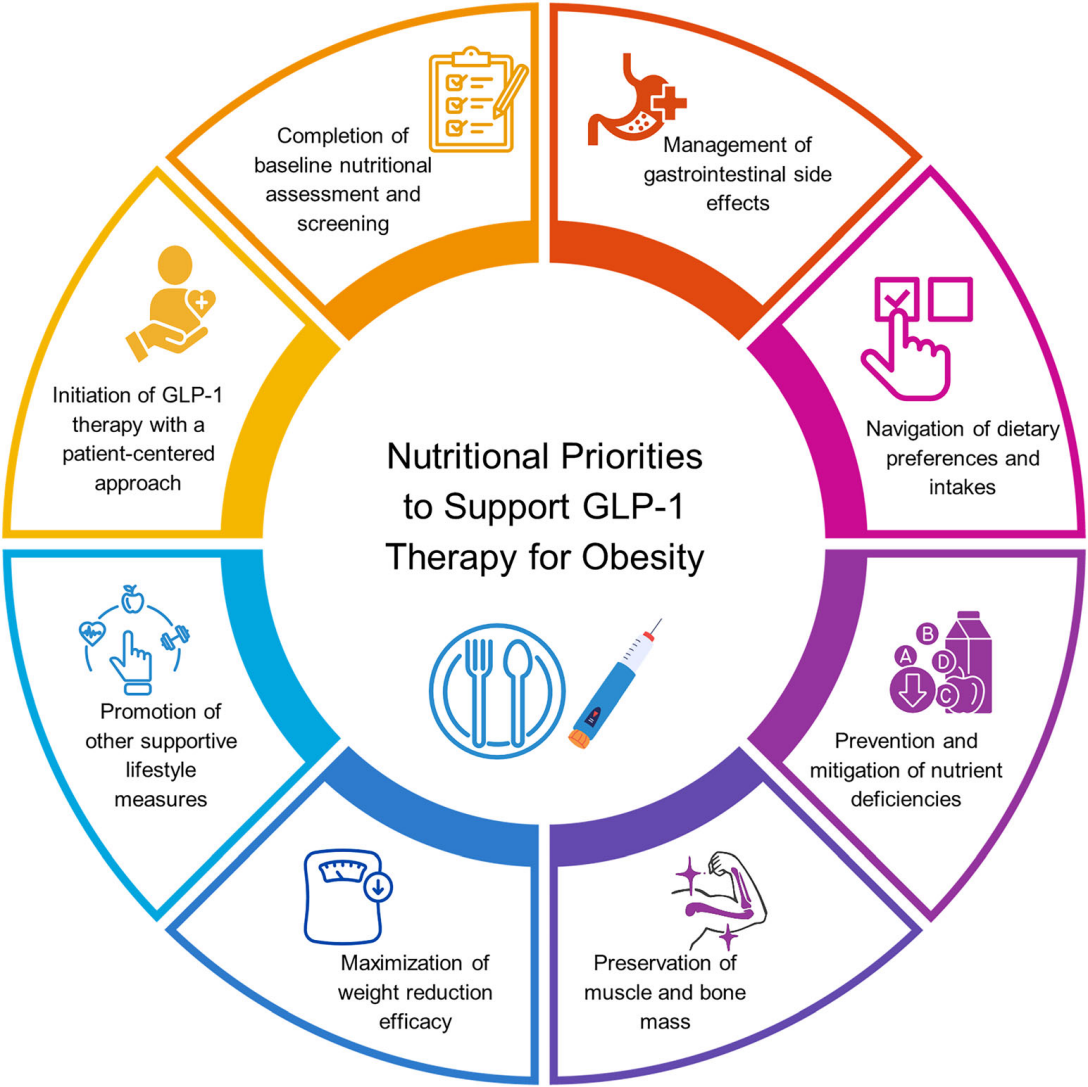
Key elements of nutritional priorities to support GLP‐1 therapy for obesity. [Color figure can be viewed at wileyonlinelibrary.com]

A pragmatic approach to nutrition and lifestyle counseling and support is recommended to maximize benefits, minimize potential risks, and increase efficiency of GLP‐1 therapy for weight reduction. The key elements are summarized in Figure 1.

### Initiation of GLP‐1 use with a patient‐centered approach

The approach to initiating pharmacologic therapy for obesity should be individualized, with a focus on overall physical health, mental health, and well‐being rather than body weight alone. Because many individuals request GLP‐1s due to a focus on body weight, other key components in the obesity management journey must be considered and discussed before initiating these therapies. A patient‐centered discussion on starting GLP‐1s should consider the individual's circumstances, preferences, values, and medical conditions. Decisions about how quickly or slowly to titrate therapy or restrict calories should be guided by an individual's needs. Some people may need to lose weight more quickly, for example those who need to qualify for surgery for a debilitating condition. Others may benefit from a slower titration schedule of medication. Screening for social determinants of health is relevant to assess potential barriers to drug access and adherence as well as lifestyle change. The 5As Framework (assess, advise, agree, assist, and arrange) is useful to guide the patient–clinician interaction to create foundation for long‐term adherence to behavior change. Table 4 summarizes the components of the original 5As framework applied to obesity care using GLP‐1 treatment.

**TABLE 4:** The 5As framework^a^ applied to support nutrition and lifestyle for obesity care using GLP‐1 therapy.

Step	Key components	Examples of topics to address
Assess	Life stage Medical history and diagnoses Physical exam, laboratory tests Food and nutrition security; dietary history and assessment Social determinants of health Psychosocial factors, e.g., mental stress, factors related to eating such as cultural and familial preferences Other potential barriers to change, e.g., food allergies or intolerances	Age of onset of problems with excess weight Periods of rapid weight gain and triggers for such exacerbations Previously attempted weight interventions (e.g., formal or informal diet or lifestyle programs, meal replacement approaches, medically monitored programs, very low‐calorie diet programs, medications, weight reduction supplements, or metabolic procedures including devices and surgeries
Advise	Benefits and risks of GLP‐1 assessment Essential complementary role of long‐term nutrition and lifestyle change Role of nutrition and lifestyle as foundations of health, with benefits beyond weight alone, with GLP‐1 as the adjunctive therapy (a re‐setting of the drug‐focused medical paradigm)	Nutritional and physical activity recommendations GLP‐1 side effects Compliance with dosing schedule
Agree	Shared plan of care to increase likelihood that both weight reduction and general health goals are understood and expectations are appropriate	Shared decision making on target body weight, and plan for continuity of care including making appropriate follow up appointments. Culturally tailored meal plan, exercise plan, etc. Including creating S.M.A.R.T. prescriptions (Specific, Measurable, Actionable, Realistic, Time‐sensitive) for eating and activity goals.
Assist	Address challenges and barriers such as food access, transportation, and need for financial resources	Eligibility assessment and enrollment support (if eligible) for federal food assistance programs such as SNAP Help find local physical activity resources such as parks, recreation centers
Arrange	Refer as needed to other specialists	Registered dietitian nutritionist Behavioral therapist Social worker Case manager

^a^ This framework can be further adapted for obesity care to begin with Ask, that is, asking permission to discuss topics such as weight and eating patterns.

## COMPLETION OF BASELINE NUTRITIONAL ASSESSMENT AND SCREENING

Prior to GLP‐1 initiation, all individuals should undergo medical and nutritional assessment and screening (Table 5) [89]. A comprehensive medical history should include details of weight history and goals and conditions that may influence nutritional needs or intake [69, 70, 119]. This includes, for example, any GI symptoms or disorders, sarcopenia, osteopenia, or osteoporosis. Individuals with a history of nephrolithiasis should be counseled to avoid high‐oxalate foods, highly processed foods, and animal‐source proteins [120, 121, 122]. Postmarket reports—which may overestimate side effects—have noted renal impairment upon initiation and dose escalation of GLP‐1s, which appears due to volume depletion resulting from dehydration caused by nausea and vomiting [123]. Persons with or at risk for renal impairment should be counseled on strategies to prevent dehydration and monitored for changes in renal function.

**TABLE 5:** Components and topics for medical and nutritional screening and assessment.

Medical history and diagnoses Age of onset of problems with excess weight Periods of rapid weight gain and triggers for such exacerbations Goals for weight reduction and general health Patient‐centered approach to consider the person's circumstances, preferences, values, and medical conditions (see Table 4)
Screening/assessment for conditions relevant to GLP‐1 use Gastrointestinal symptoms or disorders Affective disorders/mood disorders, suicidal thoughts Binge eating disorder, anorexia nervosa, bulimia nervosa, and night eating disorder^a^ Sarcopenia, osteopenia Nephrolithiasis or renal impairment
Physical exam Comprehensive clinical exam Muscle strength and function (e.g., sit‐to‐stand, stair climb, timed‐up‐and‐go; consider consultation with an exercise physiologist or strength trainer) Consider measurement of muscle mass (e.g., bioelectrical impedance analysis, air displacement plethysmography, dual‐energy X‐ray absorptiometry)
Social determinants of health Food insecurity, nutrition insecurity Housing or transportation challenges Other barriers to healthcare access
Diet history and related assessments (could be conducted by a registered dietitian nutritionist) Current dietary habits (e.g., meal/snack patterns, intake of food groups, fast foods and processed foods, cultural and household preferences) Emotional triggers for off‐plan, loss of control, or late‐night eating Food allergies, intolerances, sensitivities Conditions that may influence nutrition needs (e.g., smoking, history of kidney stones, use of certain medications) Previously attempted weight interventions (e.g., diet or lifestyle approaches or interventions, medications, or metabolic procedures including devices and surgeries)^b^
Lifestyle behaviors Physical activity including resistance training, with referral to exercise physiologist or physical therapy where appropriate Sleep habits, with referral to sleep specialist where appropriate Mental stress management, with referral for cognitive‐behavioral therapy or mindfulness‐based stress reduction where appropriate Substance use, with referral for cessation or counseling services where appropriate Social connections, consider group medical visits, shared medical appointments, weight management or peer support groups, and addressing barriers to social engagement

^a^ Persons with history of eating disorder and considering GLP‐1s for obesity should be referred to an obesity medicine specialist and an eating disorders specialist; restrictive eating disorder is a general contraindication to GLP‐1 use.

^b^ Indications that may warrant additional assessment and/or laboratory testing prior to therapy: Prior history of a very low‐calorie diet, bariatric surgery, celiac disease, other inflammatory conditions predisposing to nutrient deficiency; history of previous nutrient deficiency.

Current dietary habits should be assessed, including (1) intake of healthful foods such as fruits, vegetables, nuts, beans, whole grains, yogurt, and seafood; and (2) frequency of fast foods, frozen meals, take‐out foods, sweet and savory snacks, processed meats, and sugar‐sweetened beverages. Food allergies and intolerances, and cultural and household food preferences, are helpful to understand. A validated short screener can be useful, such as the Diet History Questionnaire [124], Mini‐EAT [125], Plant‐based dietary score [126], or Diet Risk Score questionnaire [127]. Additional assessment and/or laboratory testing may be indicated prior to therapy based on recent or current use of a very low‐calorie diet, prior bariatric surgery, celiac disease, other inflammatory conditions predisposing to nutrient deficiency, or prior nutrient deficiency [57, 128].

Clinicians should ask about positive and negative emotional triggers for off‐plan, loss of control, or late‐night eating, such as sadness, anger, boredom, or social events; and screen for affective disorders which can influence healthfulness of dietary choices and changes in calorie intake [129]. Individuals should be screened for signs of eating disorders (binge eating, anorexia nervosa, bulimia nervosa, night eating). Effects of GLP‐1s on these disorders are not well established, and could theoretically reduce or exacerbate symptoms in different circumstances. Individuals who screen positive or have a history of eating disorders should be referred to an obesity medicine specialist and an eating disorders specialist prior to prescribing GLP‐1s; restrictive eating disorder is a general contraindication [130, 131, 132].

Many people who reduce their weight on GLP‐1s experience improved mood, including fewer depressive symptoms [130, 132, 133]. While risk and causation are not established, individuals should also be screened and monitored for worsening of mood disorders or suicidal thoughts, and GLP‐1s should be discontinued if symptoms develop [134]. The role of GLP‐1s in the setting of antidepressant medications, which could have both synergistic and opposing benefits and side effects [135], requires more study.

Individuals should be assessed for risk of sarcopenia and osteopenia, seen in individuals who are older, sedentary, chronically ill, malnourished, or with type 2 diabetes. Clinicians should inquire about baseline activity levels, including strength training. For more formal quantification, validated screeners include the Physical Activity as a Vital Sign questionnaire [136] and the International Physical Activity Questionnaire [137]. For time efficiency, brief questionnaires that assess multiple lifestyle behaviors include the Lifestyle Medicine Assessment [138] and Lifestyle Medicine Health Behavior Scale [139].

To achieve the screening necessary for appropriate patient care (Table 5), efficient implementation strategies are required. For example, screening tools can be incorporated into the electronic medical record, and many could be completed by the patient through digital portals prior to their clinical visit. Additional training of providers and team‐based care are also important to ensure familiarity with these tools and their implications for care.

### Management of GI side effects

Nausea, vomiting, constipation, and diarrhea pose challenges to compliance and optimal long‐term outcomes. Because the health benefits of obesity treatment generally outweigh these temporary challenges, both proactive prevention strategies and effective support are crucial during periods of therapy adjustment. For example, gradual dose escalation helps the body adjust over time, minimizing the frequency and severity of GI symptoms [40]. During dose escalation in the clinical trials, subjects were allowed to remain at a GLP‐1 dose for up to 8 weeks, as needed, to allow GI side effects to dissipate [14, 15]. In the clinical experience of some authors of this Advisory, another approach is to maintain individuals at the lowest effective dose and escalate only as needed (i.e., when weight reduction ceases or efficacy wanes), although shortages or lack of insurance coverage of medications at lower doses may be a barrier.

Before initiating therapy, clinicians should present GI side effects in detail, advise individuals to contact them early if side effects develop and provide mitigation strategies should side effects occur. GI side effects are generally more likely to occur during GLP‐1 initiation or dose escalation. Nausea is the most common GI side effect and often occurs in the morning or after longer periods without eating. Smaller, more frequent meals and avoiding fatty or high fiber foods during the first few days of treatment can help alleviate symptoms [45]. Some individuals get caught in a cycle of not eating due to nausea, which worsens the symptoms, which then further reduces the likelihood of eating. Individuals can be counseled to eat a small breakfast and then additional small meals every 3–4 h while drinking adequate fluids. Ginger or peppermint tea, as well as acupressure bands, can be beneficial. Anti‐nausea medications can also provide relief while individuals adjust to therapy and during dose increases; agents such as prochlorperazine may be preferable to those targeting serotonergic receptors (e.g., ondansetron) that can worsen constipation. Vomiting is more likely to occur with large meals. Dehydration from severe nausea, vomiting, or diarrhea can cause acute kidney injury, with or without existing kidney disease [140], as well as heart palpitations, so efforts should be made to prevent dehydration.

Constipation is common with weight loss and should be managed proactively. Extended constipation can also lead to reactive diarrhea. Adequate fluids and fiber from foods should be encouraged, although additional strategies are often required. Foods with lower viscosity (i.e., that flow easily), fewer calories, lower glycemic index, and higher water content (e.g., certain fruits and vegetables and fruits) can facilitate faster gastric emptying [141]. Gradual increase in foods with soluble and insoluble fiber, such as prunes or other dried fruits, can be helpful. Foods high in protein or fat can further slow gastric emptying, which can promote weight reduction and metabolism but also worsen constipation, potentially requiring temporary limitation of these foods [141]. If dietary strategies are insufficient, other therapies include daily magnesium supplementation, titrated to keep bowel movements regular. Magnesium citrate is effective and well‐tolerated, and powdered forms permit customized dosing. Fiber supplements or capsules and Polyethylene Glycol 3350 may also be beneficial. Stool softeners may also be helpful in avoiding straining.

Diarrhea can also occur. Avoidance of large or high‐fat meals can be helpful. If significant diarrhea occurs, fiber capsules or powders provide bulk to the stool, and anti‐diarrheal medications can provide acute relief. Alcohol use may also worsen nausea and gastroesophageal reflux with GLP‐1 therapy and should be minimized [58].

### Navigation of dietary preferences and intakes

GLP‐1s meaningfully impact total energy intake and food preferences through multiple mechanisms—an active area of investigation—including peripherally in the gut, centrally in multiple brain regions, and through diet‐microbiome–brain interactions [142]. GLP‐1 receptors in the mesolimbic system are implicated in the modulation of reward behavior [143], whereas brain imaging studies document GLP‐1‐induced changes in brain regions related to appetite and reward, such as the insula, amygdala, putamen, and orbitofrontal cortex [144]. In experimental studies, obesity‐related hypothalamic inflammation can cause uncoupling of energy intake compared with expenditure [145]; preclinical studies suggest that GLP‐1 receptor activation may modulate inflammatory and immune responses that affect the brain [146]. Further studies are warranted to elucidate the effects of GLP‐1s on brain reward circuits and psychological dimensions of appetite and eating.

GLP‐1s reduce energy intake by 16%–39% compared with placebo, related to changes in cravings, hunger, and fullness [56, 147, 148]. Multiple studies demonstrate beneficial effects on food cravings and disordered eating. This includes reduced food preoccupation or “food noise”, reduced emotional eating, less external eating (i.e., eating that responds to external triggers, irrespective of satiety), and fewer binge eating episodes [56, 149]. Similar effects have been observed on eating control, sweet cravings, and symptoms of food addiction [147, 150, 151, 152, 153].

In addition to lower energy intake, many individuals report changes (increases and decreases) in preferences for specific foods [56]. However, these influences are less rigorously documented, with varying study results. Different studies suggest reduced cravings for savory foods and high‐fat foods [150, 151]; sweet, savory, or dairy foods [144]; salty, spicy, starchy, or dairy foods [147]; and sweets, carbohydrates, starches, and fast‐food fats [154]. The evidence supports a general preference shift away from sweet, savory, starchy, and high‐fat foods. Anecdotal reports also suggest a reduction in taste enjoyment and cravings for ultraprocessed foods and an increase in preferences and cravings for minimally processed, nutrient‐dense foods like fruits and vegetables [155]. Dietary counseling may modify these changes. For instance, in one observational study, a larger reduction in added sugars and a greater increase in dietary protein were seen among participants receiving GLP‐1s plus dietary counseling compared to GLP‐1s alone [156].

Authors in this writing group have observed in clinical practice the changes to food preferences and eating behaviors described here as follows: a substantial number of individuals are less interested in food; cravings for high‐fat foods, sugary foods, and alcohol are diminished; and binge eating, loss‐of‐control eating, and food rumination are reduced. In contrast, GLP‐1side effects such as nausea may trigger cravings for comfort foods containing sugars or refined carbohydrates such as white flour and white rice. Some report food aversions, sometimes severe, typically at the initiation of treatment and with dose increases. A limited interest in food, reduced hunger, and increased fullness may cause individuals to go several hours without eating. For some individuals, this can cause inadequate nutrient intake; for others, it may contribute to rebound preferences for sugars and refined carbohydrates if they delay eating until they are overly hungry. At times, frustration or even a loss of quality of life from the reduced pleasure obtained from food (or other aspects of life) may result in changes in effect and potentially medication discontinuation [157]. In these situations, it is beneficial to discuss with individuals whether this is related to disordered thoughts about “food as love,” affective changes induced by the medication, or a loss of interest in a food‐related hobby such as cooking [157]. Referral to behavioral therapy may be warranted. Some individuals may benefit from a change in the dose, agent, or class of obesity management medication.

### Prevention and mitigation of nutrient deficiencies

Dietary guidance for individuals using GLP‐1s should focus on ensuring nutrient adequacy within an often substantially lower‐calorie diet. To support this, clinicians should emphasize a diversity of nutrient‐dense, minimally processed foods such as fruits, vegetables, whole grains, legumes, lean proteins, nuts, and seeds. Individuals should be counseled to avoid refined carbohydrates (i.e., refined grains, flour, starches, sugars), sugar‐sweetened beverages, red and processed meats, and most fast foods, ultraprocessed sweets, and savory snacks (Table 6). Dietary supplements can be proactively considered for at‐risk nutrients, such as vitamin D, calcium, B12, or a multivitamin‐mineral tablet, at appropriate doses and tailored to each person's needs.

**TABLE 6:** Key dietary recommendations to support effective GLP‐1 therapy^a^.

Factors to encourage	Factors to minimize/avoid
Food groups	
Fruits (e.g., berries, apples, citrus fruits, banana, grapes, avocado)	Refined carbohydrates (processed grains, flours, added sugars)
Vegetables (e.g., broccoli, leafy greens, tomatoes, carrots, peas, squashes)	Sugar‐sweetened beverages
Whole grains (e.g., oats, quinoa, brown rice, and whole‐grain breads, cereals, and pastas)	Red and processed meats
Dairy (e.g., yogurt, milk, cheese)	Most fast foods
Lean proteins (e.g., poultry, fish/seafood) and eggs	Sweets and savory snacks
Nuts and seeds (e.g., almonds, peanuts, chia seeds, sesame seeds, hemp seeds)	
Plant fats/oils (e.g., olive, canola, avocado oils)	
Ginger or peppermint tea	
Eating habits^b^	
Regular, small meals at consistent times	Emotional, mindless, or nighttime eating
Flexibility with food choices	Long periods without meals (i.e., becoming overly hungry)
Enjoy portion‐controlled treats	Consumption of large meals
Ensure adequate fluids	
Minimal alcohol intake	

^a^ Nutritional recommendations and counseling are important to support weight reduction, prevent and mitigate gastrointestinal side effects, reduce muscle and bone loss, and support long‐term weight maintenance.

^b^ A registered dietitian nutritionist can help determine a dietary pattern that meets nutrition goals while accommodating an individual's dietary needs and preferences. Additional behaviors generally associated with long‐term weight maintenance include regular physical activity (≥60 min/day); self‐monitoring of body weight, food intake, and activity; limiting screen time (<10 h/week); and use of coping strategies including social support, advance planning, and problem solving skills.

Small, frequent meals may be effective when hunger and food interest are low [58]. Healthfully prepared smoothies and protein drinks with fruits, vegetables, and various unsweetened milks or yogurt; cottage cheese and soups can provide needed nutrients and are often more appealing to individuals than heavier foods such as red meats, cold cuts, or hard cheeses. If changes to food composition are not enough, setting an alarm or other reminder to eat can be helpful. Sufficient dietary protein should be a priority to help preserve muscle mass and bone density, particularly in combination with a structured strength training program (see below).

Ongoing monitoring and follow‐up should include regular re‐assessment of dietary intake and hydration, for example, using food logs and/or food photos, and re‐assessment of nutrient levels, using clinically accepted methodologies, during therapy to identify and promptly address emerging deficiencies. Dietary recommendations should be adjusted based on the rate of weight reduction, nutrient status, individual tolerance, and treatment response.

### Preservation of muscle and bone mass

The adverse effects of weight reduction on muscle and bone mass—particularly among individuals with insufficient physical activity or protein intake or at older ages [63, 158]—have highlighted the interrelated priorities to preserve muscle mass, muscle quality, bone mass, and physical function. Decreased and/or low muscle and bone mass negatively impact health, including physical impairment or disability, falls and fractures, surgical complications, reduced quality of life, and decreased survival [159, 160].

For the general adult population, the recommended daily allowance for protein is 0.8 g/kg/day [161]; this reference value is currently undergoing review for updating by the National Academies of Medicine. Higher targets, such as 1.2–1.6 g/kg/day, have also been proposed during active weight reduction [162, 163]. For individuals with obesity, it is unclear whether these goals should be based on actual body weight, corrected (adjusted or ideal) body weight, or fat‐free mass, as the use of actual weight can significantly overestimate protein requirements [164]. Protein intake in adults should not fall below 0.4–0.5 g/kg/day, as this can lead to muscle atrophy and functional impairments, whereas prolonged intake at or above 2 g/kg/day should be avoided due to potential adverse health effects [165]. Estimated fat‐free mass may be best for determining protein needs, although there is still no consensus on the optimal approach. A protein intake of 1.5 g per kilogram of lean body mass (FFM) per day is considered more accurate but requires body composition data for precise calculation [166]. Alternatively, setting an absolute protein target of 80–120 g/day, or 16%–24% energy on a 2000 kcal/d diet, may enhance adherence while ensuring adequate intake.

For individuals on GLP‐1s, adequate dietary protein may be difficult to achieve due to reduced appetite and/or taste aversions. Protein‐rich foods can be consumed first in a meal to increase the likelihood of sufficient consumption. Among food sources, plant sources (e.g., beans, peas, lentils, whole grains), dairy, seafood, eggs, and lean poultry should generally be encouraged based on their links to general health, with red and processed meats considered in moderation or minimized given links to type 2 diabetes, cardiovascular disease, and colorectal cancer in general populations [60]. Practically, lower volume, nutrient‐dense protein foods can be encouraged, such as fish, eggs, Greek yogurt, cottage cheese, and nuts/seeds, including their spreads, such as peanut or almond butter. Some individuals can meet protein targets by supplementing with high‐protein shakes, bars, and other fortified products [159].

Importantly, clinicians should understand—and emphasize to individuals taking GLP‐1s—that increased protein intake alone is likely inadequate to support the preservation of muscle mass in the absence of structured resistance/strength training. Excess dietary protein, above muscle needs for repair or growth, can be converted to fat by the liver and increase visceral adiposity [167]. Structured strength (resistance) training or mixed training (resistance plus aerobic) programs are well established to help preserve lean mass during weight reduction [62, 168]. Aerobic activity alone has a smaller effect on preserving lean mass during rapid weight reduction [168]. Retrospective studies of GLP‐1 therapy support the role of structured exercise programs, for example, 360 min/week with an emphasis on strength exercises to preserve fat‐free mass [169]. In a recent randomized trial, 1 year of combined GLP‐1 therapy with exercise training preserved bone mineral density, while GLP‐1 therapy alone decreased bone mineral density [170]. In that trial, GLP‐1 therapy plus exercise also produced larger reductions in abdominal fat and systematic inflammation than GLP‐1 therapy alone [171]. Aerobic and resistance training exercises also improve insulin sensitivity, vascular function, and oxidative stress, critical for long‐term cardiometabolic health [172].

Based on these findings, GLP‐1s should be prescribed together with a structured exercise program, aiming for regular strength training at least three times weekly plus at least 150 min of moderate‐intensity aerobic exercise weekly to preserve muscle and bone mass [62, 67, 158, 173, 174, 175]. These plans should be customized to match the individual's fitness level and physical capacity to ensure adherence and effectiveness [174, 175].

Several methods can monitor muscle mass for excessive reduction [176]. Bioelectrical impedance analysis (BIA) is pragmatic, easily implemented at point‐of‐care, and requires minimal staff training and clinic time. BIA allows for repeated measures at low cost, for instance, when weight reduction trajectories are high and muscle loss is more likely. Air displacement plethysmography can be used for individuals with pacemakers, implantable cardioverter defibrillators, or other electronic medical implants who cannot use a BIA machine—but require regular calibration, staff training, and use of close‐fitting clothing, which may be uncomfortable for some individuals. Dual‐energy X‐ray absorptiometry (DXA) scanning with body composition programming is considered a gold standard, yet it is also more costly and less likely to be done frequently. For monitoring of individuals taking GLP‐1s, DXA could be considered yearly or every 2 years, although such a timeline may impede the identification of early muscle loss and institution of appropriate interventions. Additionally, many imaging sites with DXA technology do not have or wish to use the additional body composition programming, given staff and time constraints. Newer technologies, such as visual‐based capture using a smart phone, are being developed and validated and may be more widely used in the future [177].

All these technologies monitor muscle mass but not muscle health, quality, or function. Muscle strength can be monitored in several ways. Still, some are less sensitive to change (e.g., handgrip strength) or not feasible to conduct in a clinical setting (e.g., quadricep isometric strength). Sit‐to‐stand, stair climb, and timed‐up‐and‐go measures can be helpful in older adults, but these measures may be less able to detect changes in younger individuals. While a one‐repetition maximum is a classic measure of muscle strength, it is not recommended unless the individual is highly trained. Research is underway to examine how GLP‐1 therapy affects muscle quality and physical function in younger populations, which should provide insights into appropriate imaging, functional testing, and lifestyle recommendations. Until then, consultation with an exercise physiologist or strength trainer may be beneficial for many individuals to establish general strength assessment, implement a resistance program (trainer, class, or self‐directed), and monitor over time.

### Maximization of weight reduction

A structured, comprehensive nutrition and lifestyle program could help augment the weight reduction efficacy of GLP‐1s, although findings have been mixed. In the STEP 3 trial of semaglutide combined with intensive lifestyle intervention (30 counseling visits across 68 weeks, including nutrition, physical activity, and other behavioral strategies, plus 8 initial weeks of meal replacements with liquid shakes, meal bars, or portion‐controlled meals), individuals experienced a 16% reduction from baseline in body weight (vs. 5.7% with intensive lifestyle intervention alone) [178]. In comparison, the STEP 1 trial that included semaglutide with general nutrition and physical activity instructions demonstrated a 14.9% weight reduction from baseline (vs. 2.4% with general nutrition and physical activity instructions alone) [14]. In the SURMOUNT 1 trial, tirzepatide 15 mg/week with general nutritional instructions produced a 20.9% reduction in body weight from baseline (vs. 3.1% with general nutrition instructions alone) [15], whereas in the SURMOUNT 3 trial, tirzepatide 10 or 15 mg/week started after 12 weeks of intensive lifestyle intervention produced a 25% reduction from baseline (vs. 4.8% with intensive lifestyle intervention alone) [178]. This 25% body weight reduction resulting from a staged approach with 12‐week intensive lifestyle intervention followed by tirzepatide is the largest reduction seen in GLP‐1 trials to‐date. A challenge in interpreting the impact of the lifestyle strategies in these trials is lack of standardization on how “intensive” lifestyle intervention is defined or implemented. Such interventions can vary in many key components, including the frequencies of visits; individual or group settings; in‐person, telehealth, or digital delivery; targets for food composition, calorie intake, physical activity, and other lifestyle habits; use of meal replacements; mechanisms for self‐monitoring, feedback, and peer support; efforts to maximize adherence; and overall duration.

Effects of varying dietary patterns or specific food types on maximizing weight reduction with GLP‐1s require further investigation. Based on the overall evidence around nutrition and obesity including potential impacts on metabolism, the microbiome, thermogenesis, and epigenetics, the authors of this Advisory recommend eating more minimally processed, nutrient‐dense foods and fewer starch and sugar rich ultraprocessed foods for optimizing weight reduction while using GLP‐1s (Table 6).

### Other supportive lifestyle interventions

Other lifestyle interventions are essential to support individuals using GLP‐1s as part of the recommended multicomponent lifestyle programs that are the foundation of obesity treatment [94, 179, 180]. These include improving sleep quality, managing mental stress, minimizing substance use, and nurturing positive social connections [181, 182].

Poor sleep is associated with insulin resistance, increased hunger, and weight gain, which might reduce some benefits of GLP‐1s [183]. Conversely, weight reduction achieved with GLP‐1s can improve symptoms of obstructive sleep apnea, a common obesity‐related condition [184]. Clinicians should assess GLP‐1 candidates for sleep habits using validated questionnaires like STOP‐BANG or the Pittsburgh Sleep Quality Index [185, 186]; and inquire about hypnotic drug use and insomnia [187] and symptoms of restless legs syndrome [188]. Individuals with a positive screen should be referred to a sleep medicine specialist.

Mental stress should be addressed among individuals with obesity, as chronic stress may promote obesity development through the sympathetic nervous system and hypothalamic–pituitary–adrenal axis activation that elevates cortisol levels, interferes with insulin sensitivity, promotes energy storage, and creates food cravings for ultraprocessed “comfort foods” [189] GLP‐1s may act both centrally and peripherally to reduce these downstream impacts of chronic stress and obesity and alter food reward pathways in positive ways [132]. Referral for cognitive‐behavioral therapy or mindfulness‐based stress reduction interventions may be considered for individuals receiving GLP‐1s to assist with weight reduction maintenance, glucose control, and mental stress [190, 191]. Enhanced mindfulness may also help individuals cope with GLP‐1side effects [192]. Individuals who report high levels of stress on a Perceived Stress Scale 10‐item questionnaire [193] or a brief Patient Health Questionnaire for Depression and Anxiety [194] may particularly benefit from stress mitigation interventions.

Substance use, including tobacco, alcohol, opioid, and illicit drug use disorders, should be addressed to maximize GLP‐1 benefits. Substance use and cessation have complex associations with obesity, with overlapping brain pathways with food reward and disordered eating [187, 195]. Through these interrelated pathways, GLP‐1s use may also help reduce alcohol and other substance use disorders [196]. In a recent phase 2 randomized trial, 9 weeks of low‐dose semaglutide in 48 outpatient participants with alcohol use disorder led to reductions in some but not all measures of alcohol use and craving. They led to reductions in tobacco use in the subgroup of current smokers [33]. Clinicians should educate individuals about the potential interactions between these substances and GLP‐1s and routinely screen for substance use using validated short tools such as those proposed by the Institute of Medicine (now National Academy of Medicine) for Social and Behavioral Determinants of Health [197]. If screening is positive, referral to cessation programs or counseling services can provide additional support.

Strong social connections may enhance treatment outcomes and adherence to GLP‐1s and lifestyle therapies. Robust social networks improve health outcomes by reducing stress, increasing motivation, and encouraging accountability [198, 199]. Given the network effects of obesity and the added mortality impact of social isolation/loneliness among individuals with obesity, new interventions should be studied to promote social connectivity in conjunction with GLP‐1 use [200, 201]. Clinicians can support individuals by conducting GMV or shared medical appointments (see below), recommending in‐person or virtual participation in weight management groups or peer support groups, and addressing barriers to social engagement, such as isolation or mobility challenges [202, 203].

Implementing these strategies requires a person‐centered approach, discussing these issues with each individual to understand their situation. Team‐based care, including registered dietitian nutritionists (RDNs), exercise physiologists, and health coaches, can be very helpful [204] but is not always available to clinicians or individuals depending on health system circumstances and payer policies. Pharmacists can also play a role, as an accessible healthcare professional who is also dispensing the medication. Based on the human, societal, and financial burdens of obesity, as well as the costs and adherence challenges of GLP‐1 therapy, more comprehensive weight management programs and research on their optimal use are needed in healthcare.

## BEHAVIOR CHANGE AND IMPLEMENTATION SUPPORTS

### Group‐based visits

Group medical visits (GMVs) or shared medical appointments are an increasingly established, evidence‐based modality to provide effective lifestyle therapy in a comprehensive, easy‐to‐access medium [205]. In both traditional fee‐for‐service and value‐based health delivery models, GMVs can increase access to healthcare professionals, promote in‐depth, unrushed medical visits, promote social connection and support, and improve individual engagement and outcomes [206].

Compared to conventional visits, GMVs have demonstrated improved dietary habits, improved sleep, greater patient satisfaction, better glycemic and blood pressure control among individuals with diabetes mellitus, modest but significant weight reduction improvement, particularly in females and older adults, and reduced healthcare costs [207, 208, 209, 210]. One retrospective study identified higher likelihood of prescribing obesity management medication as well as higher relative weight reduction with GMVs versus individual medical visits [211]. GMVs may help advance equitable obesity care: a retrospective study among majority Latino and low‐income households in a federally designated underserved area found greater absolute weight reduction (12 vs. 4 pounds) and meaningful weight reduction (55% vs. 11% with 7% + weight reduction) with GMVs versus individual visits [212]. Large, long‐duration lifestyle intervention randomized controlled trials have also employed group counseling sessions for participants, resulting in meaningful weight reduction [213].

Given the increasing rates of GLP‐1 prescriptions and the relatively well‐studied efficacy of GMVs in supporting lifestyle behaviors, combining the 2 may provide synergistic benefits. GMVs are covered by insurance payers, allowing broad access. Several health system initiatives are developing clinical pathways to integrate GMV models with prescribing obesity management medications [214]. As the use of GLP‐1s grows, the need for long‐term supportive health promotion (and not merely short‐term weight reduction) through lifestyle efforts will equally rise [91], and GMVs appear well suited for such efforts.

### Registered dietitian nutritionists

RDNs have important roles to play in delivering comprehensive obesity care, particularly by providing MNT to support lifestyle, pharmacological, and/or surgical therapy. MNT incorporates individualized nutrition assessment, diagnostics, therapy, and counseling to modify dietary behaviors, manage health conditions, and enhance well‐being [215]. In controlled trials, RDN‐delivered MNT modestly but meaningfully improves dietary quality, body weight, waist circumference, glycemic control, blood pressure, and blood cholesterol levels [216, 217, 218, 219]. RDNs who provide MNT for obesity care can follow evidence‐based practice guidelines [220] and earn a board‐certified specialist credential in obesity and weight management [221].

Pairing GLP‐1 use with RDN dietary counseling should support medication adherence, help prevent or manage GI side effects (particularly during medication initiation and dose increases), promote adequacy of nutrient intake, and support engagement in other behaviors (e.g., regular physical activity, adequate sleep, goal‐setting) that enhance long‐term weight management and overall health. RDNs can address dietary self‐monitoring, adjustments to food choices and meal timing, identification of minimally processed, nutrient‐dense food choices and guidance for preparation, portion control, problem solving, peer support, and goal‐setting [222].

Although limited direct evidence has evaluated use of GLP‐1s with or without RDN‐provided MNT, the SCALE [13], STEP 1 [14], SURMOUNT [15], and STEP 3 [178] trials each demonstrated substantial weight reduction by combining GLP‐1s with regular counseling sessions by RDNs or other qualified healthcare professionals (such lifestyle support was also provided to the placebo group in each trial). Compared to general practice, the more frequent and structured use of RDNs and MNT in these trials could be one reason why these trials demonstrated larger weight reductions than seen in real‐world GLP‐1 utilization for obesity.

However, private and public payer coverage for RDN‐provided MNT for obesity remains limited, preventing broad utilization in practice. For example, Medicare covers MNT only for individuals with diagnoses of diabetes, chronic kidney disease, and 36 months post‐kidney transplant, and for only 3 h during the first year of referral and 2 h annually thereafter [223]. Most state Medicaid programs have followed suit. Commercial plan coverage varies more widely and may provide MNT counseling for obesity but with annual or lifetime limits. Recent national policy efforts around payer coverage for GLP‐1s [224, 225] have elevated the importance of concurrent coverage for MNT as part of comprehensive lifestyle programming that should accompany GLP‐1 treatment. Intensive behavioral therapy provides a potential option for reimbursement of services by dietitians and other health care professionals; this service can only be billed by a primary care provider but can be delivered by a qualified health professional incident to that provider [109, 110].

### Telehealth and digital platforms

New telehealth and digital platforms provide opportunities to deliver personalized nutrition support for individuals on GLP‐1s. These tools can help address barriers posed by in‐person visits, enhance patient engagement, and promote adherence to nutritional and lifestyle recommendations [226, 227, 228, 229]. Relevant applications and features include video visits, collaborative care, remote patient monitoring, dietary tracking and guidance, education and behavioral support, and increased equity and accessibility (Table 7). There is hope that benefits may include improved accessibility and engagement with individuals, better tracking of progress and self‐monitoring, broader reach to underserved areas, and improved cost‐effectiveness compared to conventional nutrition support.

**TABLE 7:** Telehealth and digital platform support for nutrition during GLP‐1 treatment.

Application or feature	Example of opportunity to support nutrition
Video visits	Synchronous video visits with physicians, other practitioners, registered dietitian nutritionists (RDNs), exercise physiologists, and clinical psychologists can support both initial evaluation and for ongoing follow‐up. Such visits can improve adherence and motivation; identify, address, and provide timely feedback to manage side effects; adjust therapy including medication and goals of care; determine when in‐person visits are indicated for testing or other evaluation; and increase appointment attendance rates and patient engagement.
Remote patient monitoring	Digital engagement enables timely feedback to healthcare providers to adjust recommendations based on patient progress and feedback. Features that support remote monitoring include Bluetooth‐enabled scales, blood pressure cuffs, and continuous glucose monitors; apps with food logs and photo reviews for RDNs or other nutrition specialists; physical activity and sleep monitors; and private messaging with clinicians.
Dietary guidance and tracking	Dietary‐tracking apps can help primary care, specialty obesity care, and telehealth medical groups and platforms monitor nutritional habits against individualized nutritional goals. Specialized features such as automated nutrient deficiency alerts, AI‐powered meal recommendations, and integration with wearables can help identify nutritional gaps in real time.
Education and behavioral support	Virtual education modules and coaching sessions can teach individuals how to incorporate minimally processed, nutrient‐dense foods, manage gastrointestinal side effects, and implement sustainable dietary habits. These can further reinforce SMART (specific, measurable, achievable, realistic, timely) goals to ensure optimized nutrient therapy, physical activity, and behavioral modification alongside medical monitoring. Modules and coaching can be both synchronous and asynchronous, increasing flexibility for both clinicians and patients.
Equity and accessibility	Telehealth and digital platforms reduce geographic and other logistical barriers to quality care, for example for individuals who live in rural or underserved areas or have limited time, physical mobility, or transportation, which can reduce health disparities.

Abbreviations: AI, artificial intelligence; RDN, registered dietitian nutritionist; SMART, specific, measurable, achievable, realistic, timely.

Challenges to using these platforms include the potential for limited device or internet access; low health or digital literacy; visual, hearing, or cognitive impairment; lower emotional connection with providers; and exacerbation of social isolation. Individuals may also have limited support (family, caregivers) to assist with telehealth consultations and reluctance or lower trust to embrace unfamiliar healthcare methods. Tailored solutions can help address these challenges, which require healthcare provider knowledge and sensitivity and identification of individuals more likely to experience digital challenges (e.g., older adults) [230].

While these programs offer new ways to engage more regularly with individuals on GLP‐1s, obesity management frequently involves assessing and addressing complex behavioral, emotional, and social, for which virtual visits may not always be adequate. Several for‐profit telehealth and digital companies are now engaging with health systems, aiming to provide more efficient and less costly obesity management. Given promise as well as challenges, more research is needed on telehealth and digital interventions for adherence to GLP‐1 therapy, long‐term weight management, and individual behaviors, health outcomes, quality of life, and satisfaction.

### Food is medicine

Food is Medicine (FIM) programs are structural interventions in healthcare that offer food‐based nutritional therapies as part of an individual's plan to manage or treat specific disease conditions and, often, social needs [231]. These are prescribed by a clinician, tailored by an RDN to relevant medical conditions, and covered by health insurance. FIM therapies include medically tailored meals, medically tailored groceries, and produce prescriptions, each accompanied by varying types, delivery modalities, and intensities of nutrition and culinary education. Supportive measures include electronic health record screening for food and nutrition security, curricular and accreditation interest in medical nutrition education, and expanded care pathways and reimbursement models [231]. State Medicaid programs, Medicare Advantage payers, commercial payers, the Veterans Health Administration, and the Indian Health Service are all implementing and evaluating various FIM programs. Piloting coverage has been proposed but not implemented in traditional Medicare [232].

Evidence from pre/post, quasi‐experimental, and some randomized interventions supports the benefits of FIM for food security, nutrition security, diet quality, blood glucose control, hypertension, disease self‐management, self‐perceived physical and mental health, and health care utilization [231]. In non‐randomized interventions, FIM therapies are associated with BMI reductions of 0.4 to 0.6 kg/m^2^. However, these programs did not focus on weight reduction or exercise, suggesting that a FIM program that is expressly designed for weight reduction and maintenance could be more effective. The role of FIM for weight management, including as a potential adjunctive therapy to GLP‐1 use, is an area of growing interest and investigation. Because FIM programs can help overcome multiple barriers to healthful eating, including cost, time, access, and knowledge, they could play an important role in achieving better as well as more equitable short‐ and long‐term outcomes with GLP‐1 therapy—a critical area for further investigation.

## GLP‐1S AND HEALTH EQUITY

Health equity can be defined as a state in which everyone has a fair and just opportunity to attain their highest level of health [233], and health disparity is a particular health difference linked with economic, social, or environmental disadvantage, often adversely affecting groups of people who have systematically experienced greater social or economic obstacles. Poor nutrition and obesity disproportionately affect individuals with lower socioeconomic status in rural communities and in racial and ethnic minoritized populations [234], and disparities in poor nutrition and obesity prevalence are mirrored in disparities in access to quality health care [235].

### GLP‐1 access

Disparities in access to GLP‐1s have been documented by race/ethnicity and socioeconomic status [236]. In a study of ~1.2 million commercially insured U.S. individuals with diabetes from 2015 to 2019, lower GLP‐1 use was seen among Asian, Black, and Hispanic, compared with White, individuals and among those living in lower versus higher income households [237]. Using electronic health record data from 6 U.S. care delivery systems from 2014 to 2022, American Indian/Alaska Native, Asian, Black, Hawaiian or Pacific Islander, and Hispanic individuals were less likely to be prescribed a GLP‐1 than White individuals [238].

In comparison, while half (51%) of U.S. adults meet FDA eligibility for semaglutide using nationally representative data, this is higher among Black (57%) and Hispanic (55%) adults [239]. Yet compared with eligible White individuals, larger proportions of Black and Hispanic individuals have potential barriers to GLP‐1 access, such as being uninsured, lacking a regular provider, having low income, or lacking higher education [239]. Racial/ethnic disparities in weight bias and stigma have also been documented in healthcare settings and may affect GLP‐1 access; research and clinical opportunities to address weight stigma and foster health equity have been proposed [240]. Given unequal payer coverage, the income also directly reduces GLP‐1 access due to the inability to afford high co‐payments or self‐payment.

In a review of racial and ethnic differences in obesity outcomes of lifestyle, surgical, and pharmacological interventions published between 2000 and 2022, lower efficacy of lifestyle and surgical interventions was commonly identified among Black compared with White participants (with no or smaller differences for Hispanic compared with White participants). Findings were more mixed for pharmacologic obesity interventions, with inconsistent or no differences observed by race/ethnicity [241]. In trials, medication treatments may be more standardized and less influenced by sociocultural variables than lifestyle and surgical interventions. However, all treatment pathways showed racial/ethnic disparities in referral rates, access, engagement, and retention.

Given the importance of structured screening, monitoring, and long‐term nutritional and lifestyle support for GLP‐1s, treatment and outcome disparities will likely be further magnified by disparities in access to and use of these supports. Thus, equitable coverage for such efforts is important. Future research is needed to identify the social, environmental, structural, and other factors that influence access to GLP‐1s and supportive nutrition‐focused lifestyle programs.

### Food and nutrition insecurity

Food security and nutrition security may influence efficacy of GLP‐1s. Poor nutrition while on therapy can exacerbate GI side effects, nutrient deficiencies, and reduction of muscle mass; negatively impact metabolic health and risk of chronic conditions; and reduce likelihood of long‐term weight maintenance (especially if GLP‐1 therapy is stopped). Food insecurity is closely tied to poverty and financial strain, measured as a household‐level economic and social condition of limited or uncertain access to adequate food [242]. In 2023, 13.5% of U.S. households were food insecure [243], with prevalence varying by race/ethnicity, family income, educational attainment, and disability status [244].

As compared to food security measures which assess regular access to sufficient food (quantity or calories), nutrition security is a distinct but related concept that evaluates consistent access, availability, and affordability of foods and beverages that promote well‐being and prevent and, if needed, treat disease [245]. Validated screening tools such as the Nutrition Security Screener have been implemented in large health systems, regional, and national surveys and identified the prevalence of nutrition insecurity as modestly higher than that of food insecurity, with only partial overlap (correlations: ~0.4 to 0.6), highlighting the distinction between access to sufficient calories versus nourishing foods [246]. Commonly reported barriers to healthy eating included cost (81%), lack of knowledge on how to cook healthy foods (75%), too few healthy foods at local stores (53%), or long distances to healthy food stores (46%); lack of healthy cultural foods (49%); and insufficient equipment to prepare (41%), time to shop for (41%), or time to cook (34%) healthy foods. Nutrition insecurity was more common among adults with younger age, lower income, lower educational attainment, and identifying as Black, Hispanic, or Native American/indigenous compared with White backgrounds [246]. Adjusting for age, sex, race/ethnicity, income, education, and food security status, individuals experiencing nutrition insecurity were 40%–60% more likely to have obesity as well as type 2 diabetes, heart disease, hypertension, and hypercholesterolemia. In contrast, adjusting for sociodemographics and nutrition security status, individuals experiencing food insecurity were not more likely to have obesity.

Food sovereignty—“the right of peoples to healthy, culturally appropriate food produced through ecologically sound and sustainable methods, and their right to define their own food and agriculture systems”—is also relevant to equitable GLP‐1 outcomes [247]. For example, food sovereignty among indigenous North American communities can be promoted through community ownership, inclusion of traditional food knowledge, use of culturally relevant foods, and environmental sustainability [248, 249]. Tailoring GLP‐1‐coupled nutrition interventions to promote food sovereignty may help reduce disparities in diet‐related diseases that persist among historically minoritized groups with strong cultural food traditions [250, 251].

The inability to consistently access sufficient and nutritious food affects an individual's ability to adhere to an obesity treatment plan, particularly given the importance of dietary modifications to optimize the benefits of GLP‐1 use, reduce nutrient shortfalls, and manage side effects. The identified challenges underscore the need for supportive clinical and population policies that equitably address food and nutrition insecurity to support effective, cost‐effective, and equitable use of GLP‐1s. Such strategies could include, for example, greater integration of FIM programs into clinical care, strengthening of federal nutrition assistance programs to address food and nutrition security, and regulatory policies to address the quality of foods available to the public [252].

### Nutrition and culinary knowledge

Nutrition knowledge and culinary skills are associated with the health profile of dietary choices [253, 254, 255]. Decreased emphasis on nutrition and culinary education in public schools; changing household and sociocultural family dynamics around eating; increased development, accessibility, and marketing of low‐cost ultraprocessed packaged foods; and higher perceived financial and opportunity costs of many health‐promoting foods have each contributed to a dearth of nutrition and culinary knowledge and competencies [256, 257, 258]. These factors have particularly impacted individuals with lower health literacy, food/nutrition literacy, and socioeconomic status—perpetuating health inequities for diet‐related diseases [256, 259]. Clinicians are not immune to these societal trends and often lack sufficient knowledge about food, nutrition, and healthy food preparation.

Given the importance of adequate nutrition before, during, and after GLP‐1 use, clinicians and individuals must be equipped with knowledge and skills around healthy eating. Interest in medical nutrition education for clinicians is growing throughout training and practice [260, 261]. Teaching kitchen curricula for clinicians and individuals has emerging evidence for enhancing nutrition knowledge, culinary skills, and dietary pattern change [262, 263, 264]. When culturally tailored, nutrition education further supports dietary change [265, 266, 267].

## FUTURE DIRECTIONS

As clinical and public interest in GLP‐1s accelerates, the pace of peer‐reviewed evidence has not kept up to provide answers to all relevant topics. We herein highlight some timely gaps and recommendations based on limited available evidence and expert opinion to help guide clinicians in addressing real‐world questions raised in practice. With the accumulation of more science, guidance on these topics may evolve.

### Dietary modulation of GLP‐1 release and action

The GLP‐1 hormone is naturally released in response to eating by intestinal enteroendocrine L‐cells, present throughout the intestines and especially the distal colon. After eating, GLP‐1 blood concentrations rise by 2‐ to 4‐fold, activated by neuroendocrine pathways (with onset 10–15 min after eating) and nutrient‐specific G‐protein coupled intestinal receptors (with onset 30–60 min after eating) [268]. Circulating endogenous GLP‐1 has a half‐life of 1–2 min, being rapidly inactivated by dipeptidyl‐peptidase IV. Despite this, endogenous GLP‐1 exerts powerful metabolic effects, including on pancreatic beta cells and the brain [268]. The latter includes both central homeostatic (energy‐intake‐focused) and non‐homeostatic (reward‐focused) regulation of food consumption in the hypothalamus and nucleus of the solitary tract [268, 269, 270, 271], influenced by a relatively small amount of GLP‐1 that crosses the blood–brain barrier and, more notably, GLP‐1 modulation of vagal afferent neurons [268]. This latter pathway may have potent metabolic effects, even when endogenous GLP‐1 blood concentrations are lower than pharmacologic GLP‐1 levels [268].

Physiologic GLP‐1 secretion is a complex, incompletely understood process, with early studies suggesting potentiated release through nutrient supplementation, whole foods, dietary patterns, and microbiome alterations [268, 272]. In the small intestine, monosaccharides directly drive GLP‐1 secretion by binding to enteroendocrine L‐cell glucose transporters [268, 273]. In the colon, unabsorbed monosaccharides, polysaccharides, and non‐digestible carbohydrates (fiber, resistant starch) are metabolized by bacterial fermentation into short‐chain fatty acids [268, 272] that bind free fatty acid receptors 2 and 3, resulting in GLP‐1 secretion. Similarly, mono‐ and polyunsaturated long‐chain fatty acids strongly stimulate GLP‐1 release via free fatty acid receptors 1 and 4. Protein‐induced GLP‐1 secretion is poorly understood but appears to play a role in GLP‐1‐mediated satiety [268]. In experimental studies, specific bioactives also stimulate GLP‐1 secretion, for example, polyphenols in fruits and vegetables, catechins in green tea, curcumin in turmeric, capsaicin in chili peppers, omega‐3 fatty acids in fish, and cinnamon and ginger [274, 275].

Nutrient supplementation with fiber, resistant starch, and unsaturated fats are the most studied supplements in both animal studies and small‐scale human trials—often showing increased circulating GLP‐1 concentrations and associated metabolic improvements [269]. Mixed‐nutrient meals higher in protein or fiber and specific dietary patterns (e.g., Mediterranean diet) may also increase GLP‐1 secretion. Studies of microbiome alterations and GLP‐1 release are inconclusive, but may be more impactful when including both prebiotics and probiotics [272]. Further investigation is critical to understand the health implications of specific nutritional and microbiome interventions on endogenous GLP‐1 secretion. Questions include dose‐specific effects of single nutrient, food, and dietary pattern interventions; targeting multiple enteroendocrine pathways simultaneously; potential differences in subgroup responses (e.g., with insulin resistance or obesity); and effects of prebiotic, probiotic, and symbiotic therapies [268, 272].

### Improving long‐term adherence

While persistent GLP‐1 therapy is recommended for obesity control and weight maintenance, most individuals prescribed GLP‐1s for obesity treatment stop taking the drug within 1 year. Although demographic and clinical predictors of discontinuation have been assessed [74], the underlying reasons for discontinuation remain poorly quantified. In the authors' clinical experience, some individuals have challenges with side effects, out‐of‐pocket costs, or changes in payer coverage. Others tolerate the drug but, once meaningful weight reduction is achieved, do not wish to stay on the medication for life.

Adherence challenges are not unique to GLP‐1s. Nearly 1 in 3 prescribed medications are never filled, and individuals regularly adhere to only half of prescribed agents [276]. Evidence‐based strategies to improve general medication adherence include dose simplification, patient education, electronic reminders, reduced out‐of‐pocket costs, and patient incentives [5, 276]. Integrating GLP‐1 use with longitudinal, structured nutrition and lifestyle programming might also support simplified dose titration schedules and management of side effects. These interventions could be coupled with electronic reminders and FIM benefits, such as medically tailored groceries or produce prescriptions, to encourage medication adherence in the setting of dietary pattern changes. Standardized clinical workflows that incorporate structured programs of stepped therapy, supported by nutritional and lifestyle interventions, could also help promote more effective and cost‐effective use for individuals and healthcare systems.

Importantly, adherence with dietary and other lifestyle changes is also challenging for many people. Just as occurs following GLP‐1 discontinuation, weight regain is common with waning adherence to dietary and physical activity weight loss interventions. Approaches to address and support the ability of individuals to achieve long‐term success with their overall weight management program are a critical area for future implementation research.

### Nutritional priorities for long‐term weight maintenance post‐therapy

Given adherence challenges, clinicians should help individuals establish positive nutrition and other lifestyle practices before and during GLP‐1 therapy, to increase success at maintaining such practices if the drug is stopped. Weight maintenance is one top goal—preserving health gains as much as and for as long as possible. While the specific nutritional and other behaviors contributing to weight maintenance post‐GLP‐1 therapy have not been rigorously studied, other observational data elucidate general predictors of successful long‐term weight reduction. For example, the National Weight Control Registry has identified several nutrition‐related correlates of weight maintenance [277, 278], including eating at regular times daily; eating regular breakfast; eating more minimally processed foods higher in nutrients, fiber, and/or protein; avoiding sugary drinks, highly processed foods, and snack foods; and permitting flexibility with food choices and occasional portion‐controlled treats rather than severe restriction. Other behaviors associated with success include regular physical activity (≥60 min/day), self‐monitoring of body weight, food intake, and activity, limiting screen time (<10 h/week), and use of coping strategies including social support, advance planning, and problem solving skills.

### Combination or staged GLP‐1 with nutrition therapy

Given the existing adherence and cost challenges of GLP‐1s coupled with significant weight regain after discontinuation, combination or staged therapy with intensive lifestyle management could promote greater efficacy, cost‐effectiveness, and equitable obesity care [3, 18, 20, 231, 279, 280]. Both the STEP‐1 and SURMOUNT‐4 trials included the availability of RDN dietary counseling and recommendations to exercise 150 min/week during the treatment period, but with notable weight regain for most participants upon GLP‐1 discontinuation without continued nutrition support [18, 20]. In the SURMOUNT‐3 trial, individuals who received tirzepatide after a comprehensive 12‐week lifestyle intervention achieved a mean 25% weight reduction, compared to 4.8% in the placebo group [178], suggesting a benefit for staged lifestyle intervention with GLP‐1s. Overall, preceding or combining GLP‐1s with intensive behavioral therapy shows promise in increasing achieved weight reduction. Notably, such programs did not include a full spectrum of evidence‐based behavioral therapies—such as tailored support and tracking for nutrition, culinary skills, physical activity, sleep, stress management, social connectivity, and medication management—which could further augment efficacy [181, 182].

Given high rates of discontinuation, use of intensive lifestyle management could also aid in weight maintenance long term. A recent simulation analysis compared continuous GLP‐1 therapy versus a staged program of GLP‐1 therapy until sustained weight reduction was achieved, followed by discontinuation and a structured behavioral lifestyle intervention for weight maintenance [84]. With a wide range of plausible effectiveness and costs of behavioral intervention, this alternative program was projected to generate substantial savings in net healthcare costs, with minimal loss of health‐related quality of life. Such programs will unlikely work for all or even most individuals. However, given high GLP‐1 costs and discontinuation rates, success among even a modest proportion of individuals could greatly augment overall efficacy and cost‐effectiveness of GLP‐1 therapy.

Nutrition counseling may be insufficient for individuals who face significant barriers to healthy eating, such as food insecurity, nutrition insecurity, or insufficient time or income. Incorporating FIM interventions, such as medically tailored groceries or meals, may improve compliance with nutrition recommendations during GLP‐1 use and, if stopped, thereafter [3]. Consistent with this, in non‐GLP‐1 weight reduction studies, access to healthy food at home is associated with weight maintenance [280]. The current challenges and costs of GLP‐1 therapy highlight the urgent need for rigorous research on how staged or combination nutritional programs, including multiple components and possibly FIM therapies, might improve outcomes, mitigate weight regain or cycling, and promote health equity.

### Nutritional considerations for off‐label use

Some individuals use “microdosing”, spaced out dosing, or lower compounded dosing of GLP‐1s. Such use may be motivated by personal preferences, GI tolerability, costs, and drug shortages. Cost‐related drug rationing is more common among those who are younger, female, lower income, uninsured, or have prevalent obesity or cardiovascular disease (CVD) [281]. Analyses of social media suggest that off‐label use is influenced by dosing concerns, insurance denials, and GI side effects [282, 283, 284]. Off‐label use can lead to dosing errors and reduced efficacy. Concerns have been raised about off‐label use of compounded GLP‐1s [285], including for cosmetic weight reduction [286, 287, 288, 289]. Nutritional considerations for off‐label use remain unclear and, given the rapidly growing public use of GLP‐1s, are an important area of needed research.

### Use of specific dietary patterns

Several dietary practices and topics of public interest intersect with use of GLP‐1s, including ketogenic diets, intermittent fasting, and ultraprocessed foods. Ketogenic or very‐low‐carbohydrate diets can be a practical approach to weight loss and glycemic control for some people, while others find long‐term adherence difficult [290, 291, 292]. People on ketogenic diets should be counseled to prioritize minimally processed foods, those with higher fiber, such as vegetables, and nutrient‐dense foods to ensure nutritional adequacy (Table 6). For individuals with diabetes, the ketogenic diet in combination with GLP‐1 therapy could increase the risk of diabetic ketoacidosis and hypoglycemia. Discontinuation or reduction of sulfonylureas and insulin should occur with careful monitoring by the primary care or provider, as appropriate, for individuals with type 2 diabetes interested in a ketogenic diet [293, 294].

Intermittent fasting may increase the risk of hypoglycemia in individuals with type 2 diabetes on hypoglycemic agents and those with type 1 diabetes [295, 296]. Individuals on GLP‐1 therapy may also practice unintended intermittent fasting, due to not being hungry. Even when using intermittent fasting, individuals taking GLP‐1s should be encouraged to consume meals at regular times of the day. Long periods of fasting without sufficient protein intake or dietary variety can lead to nutritional inadequacy, clinical nutrient deficiencies, loss of fat‐free mass, and reduced resting energy expenditure [297]. These effects can be mitigated through strength training, adequate protein and calories consumption, and a variety of minimally processed, nutrient‐rich foods (Table 6) [298].

Ultraprocessed foods are defined by the use of industrial additives or processing technologies not normally used in home cooking [299]. Mechanisms of harm appear likely varied and could include changes to the food matrix; higher starch, sugar, or salt; less fiber, micronutrients, or polyphenols; harms of certain additives, industrial toxins, or packaging contaminants; and displacement of minimally processed, healthful foods [299]. Avoiding these foods is generally advisable, although certain subcategories of ultraprocessed foods may have neutral or net positive health effects (e.g., those rich in whole grains, fruit, yogurts, or seafood), depending on their ingredients, processing, and additives [300, 301].

### Definitions and diagnostic criteria for clinical obesity

A recent expert group reviewed the utility of BMI‐based measures for assessing individual health and concluded that these can misclassify (both underestimate and overestimate) adiposity—and thus undermine effective clinical care and policy development [271]. To address this, the report proposed a new definition of *clinical obesity*—a chronic, systemic illness resulting from excess adiposity and characterized by alterations in tissue and organ function. The report further distinguished clinical obesity, defined as excess adiposity with significant tissue or organ dysfunction that can lead to severe complications, from preclinical obesity, defined as excess adiposity without immediate organ dysfunction but with an increased risk of progression to clinical obesity and other non‐communicable diseases.

That expert group recommended using BMI as a surrogate measure of clinical obesity for population‐level assessments. However, for individual health evaluations, they recommended assessing adiposity using direct body fat measurements or additional anthropometric criteria, and evaluating tissue or organ dysfunction using laboratory measurements or based on significant limitations in daily activities due to obesity. The report noted that individuals with clinical obesity should receive timely, evidence‐based treatment to improve or remit symptoms and prevent further complications; while those with preclinical obesity could be managed with health counseling and monitoring to mitigate progression.

This new proposed paradigm more closely aligns with clinical goals and practice around obesity care. How and when it may be integrated by clinical societies and practicing clinicians, as well as the impact on GLP‐1 utilization and monitoring, is an area for future investigation.

In conclusion, although GLP‐1s alone can produce significant weight reduction and related health benefits, several challenges limit its long‐term success for individuals and populations. These include GI side effects; risk of nutrient inadequacies, muscle, and bone loss; high costs; frequent discontinuation; and weight regain. Careful attention to evidence‐based nutritional and behavior modification can help mitigate the adverse effects of these challenges. Thus, all clinicians prescribing GLP‐1s for obesity management should establish a thoughtful plan of care that includes thorough nutritional and lifestyle counseling before, during, and after the weight reduction period. This should include an emphasis on healthful eating, physical activity, and resistance training; screening and management around substance use disorders, eating disorders, mental health, and sleep; and micronutrient or protein supplementation as needed. These approaches can provide benefits beyond body weight alone: reducing GI side effects, micronutrient deficiencies, and muscle and bone loss, and improving general metabolic health and well‐being. Such comprehensive care will make clinicians more effective stewards of these medications and positive contributors to their patients' health.

## SUMMARY TAKEAWAY MESSAGES

- Despite the ability of GLP‐1s to produce significant weight reduction and related health benefits, challenges such as GI side effects, risk of nutrient inadequacies, loss of muscle and bone mass, high costs, frequent discontinuation, and weight regain limit the use of these drugs for long‐term success in individuals and populations.
- Clinicians prescribing GLP‐1s for obesity management should focus on and help mitigate these challenges by developing care plans that include thorough nutritional and lifestyle counseling before, during, and after the weight reduction period.
- Such comprehensive care will support treatment benefits beyond body weight alone and will make clinicians more effective stewards of GLP‐1s and, ultimately, of patients' overall health.

## AUTHOR CONTRIBUTIONS

All authors contributed to conception and design, analysis and interpretation of the evidence, drafting of the manuscript, finalization of the manuscript, and the decision to submit to publication.

## FUNDING INFORMATION

This work was supported by the National Institutes of Health (2R01 HL115189, DM; and P30 DK04056, UE5 DK137285, and U24 DK132733, FCS) and the National Clinician Scholars Program at the Duke Clinical and Translational Science Institute (RK). The funders had no role in the conception, design, analyses, interpretation, manuscript drafting, or decision to submit the manuscript. The content is solely the responsibility of the authors and does not necessarily represent the official views of any of the funders.

## CONFLICT OF INTEREST STATEMENT

Dr. Mozaffarian reports research funding from the National Institutes of Health, Kaiser Permanente Fund at the East Bay Community Foundation, National Association of Chain Drug Stores Foundation, Google Health, and The Rockefeller Foundation; scientific advisory board, Beren Therapeutics, Brightseed, Calibrate, Elysium Health, Filtricine, HumanCo, Instacart Health, January Inc., WndrHLTH; scientific consulting, Amazon Health; equity in Calibrate and HumanCo; and chapter royalties from UpToDate. Dr. Alexander reports advisory board for Novo Nordisk and speakers bureau for Eli Lilly. Dr. Apovian reports research funding from PCORI and GI Dynamics, Inc.; and advisory boards for Altimmune, Inc., Arrowhead Pharmaceuticals, Inc., BioAge, Biolinq Incorporated, Caribou Biosciences, Inc., CinFina Pharma, Inc., Covidien LP, Cowen and Company, LLC, Currax Pharmaceuticals, LLC, EPG Communication Holdings Ltd., Form Health, Inc., Fractyl Health, Inc., Lilly USA, LLC, L‐Nutra, Inc., Mediflix Inc., NeuroBo Pharmaceuticals, Inc., Neurocrine Biosciences, Inc., NodThera Limited, Nutrisystem, OptumRx, Inc., Pain Script Corporation, Palatin Technologies, Inc., Pursuit By You, Redesign Health Inc., ReShape Lifesciences Inc., Riverview School, Roman Health Ventures Inc., Scholar Rock, Inc., Terns, Inc., Verily Life Sciences LLC, Veru Inc., Vida Health, Inc., Wave Life Sciences, Xeno Biosciences and Zyversa Therapeutics, Inc. Dr. Butsch reports advisory boards for Eli Lilly, Novo Nordisk, Abbott and Boehringer Ingelheim. S. Christensen reports advisory boards and speakers bureau for Novo Nordisk and speakers bureau for Eli Lilly. Dr. Kane reports research funding by the Ardmore Institute of Health. Dr. Stanford reports research funding from the National Institutes of Health; and scientific advisory boards for Eli Lilly, Novo Nordisk, Amgen, Pfizer, Currax, Calibrate, Vida Health, Ilant Health, Mellicell, Sweetch, Doximity, GoodRx, Empros Pharma, Clearmind Medicine, and Apnimed. The other authors report no disclosures.

## ETHICS STATEMENT

Responsibility for editorial decisions and peer review process for this article was delegated to non‐author Editors or non‐author Associate Editors.
